# Correlation between Virtual Screening Performance and Binding Site Descriptors of Protein Targets

**DOI:** 10.1155/2018/3829307

**Published:** 2018-01-11

**Authors:** Jamal Shamsara

**Affiliations:** Pharmaceutical Research Center, Pharmaceutical Technology Institute, Mashhad University of Medical Sciences, Mashhad, Iran

## Abstract

Rescoring is a simple approach that theoretically could improve the original docking results. In this study AutoDock Vina was used as a docked engine and three other scoring functions besides the original scoring function, Vina, as well as their combinations as consensus scoring functions were employed to explore the effect of rescoring on virtual screenings that had been done on diverse targets. Rescoring by DrugScore produces the most number of cases with significant changes in screening power. Thus, the DrugScore results were used to build a simple model based on two binding site descriptors that could predict possible improvement by DrugScore rescoring. Furthermore, generally the screening power of all rescoring approach as well as original AutoDock Vina docking results correlated with the Maximum Theoretical Shape Complementarity (MTSC) and Maximum Distance from Center of Mass and all Alpha spheres (MDCMA). Therefore, it was suggested that, with a more complete set of binding site descriptors, it could be possible to find robust relationship between binding site descriptors and response to certain molecular docking programs and scoring functions. The results could be helpful for future researches aiming to do a virtual screening using AutoDock Vina and/or rescoring using DrugScore.

## 1. Introduction

Molecular docking is a method in which it is attempted to find the most probable pose of the ligand in the active site of a receptor and estimation of the binding energy. Molecular docking is a computational approach whose applicability in virtual screening was approved. Comparing with experimental methods of HTS (High Throughput Screening) it can save time and cost of a drug discovery project. However, it suffers from some drawbacks such as a high rate of false positives [1, 2]. It was shown that docking programs have a reasonable power to predict correct binding pose of the ligands. However, their scoring powers were not same for different protein families and also there is a weak correlation between docked scores and binding affinities of the ligands [3, 4].

One of the most cited open source docking engines is AutoDock Vina [5]. It uses genetic algorithm to search for the most energy favorable pose of a flexible small molecule in either a rigid or a flexible binding site of a protein. Here, AutoDock Vina was employed as a docking engine. Generally, the docking engines use scoring functions to discriminate between favorable and unfavorable binding poses of the same molecule [6]. Furthermore, scoring functions rank the best binding poses of the different small molecules to find strong binders among them. The scoring functions deal with a trade-off between speed and accuracy. Thus, rescoring and consensus scoring approaches have been investigated to discover a stable method that possibly could add up the accuracy of various scoring functions and outperform single scoring functions [7–11]. However, it has been suggested that the scoring functions performances are target dependent. However, the present study is different in some aspects. The data set is retrieved from DUD-E [12] data set to avoid bias in the design of active groups and decoys data set for each protein target. In addition, the protein targets data set is diverse and we attempted to find possible relationships between scoring function performances and the binding site descriptors.

One of the proposed solutions that possibly could improve the virtual screening results is rescoring. Scoring functions can fall into three categories [6, 13]: (1) empirical scoring functions, including ChemScore [14], (2) knowledge-based potentials, including DrugScore [15], and (3) force-field based approaches, including AutoDock Vina [5] and AutoDock 4.2 [16]. Four metrics can be employed to assess the performance of a scoring function: the scoring power, ranking power, docking power, and screening power [6, 17]. Thus, rescoring can be done to find the best conformation of a single molecule (improvement of docking power) and for improvement of estimation of the binding energy and ranking the ligands (scoring and ranking power) or reranking the hits of a virtual screening to discriminate between decoys and true binders (improvement of screening power). The latter is the main concept of this research. A consensus scoring method so-called rank-by-number that had shown promising results [9] was also tested in this study. Several reports [1, 7–11] investigated the possible effects of rescoring on the different metrics of scoring performance. Among them the main result of more recent studies that have been done on larger data sets is that scoring function performance is very dependent on target [1]. In the other words, the current scoring functions are not universal.

In this study it was attempted to evaluate rescoring performance in virtual screenings conducted on a large set of predefined ligands and decoys for 32 receptors. In addition, the aim of this study is to find a method to predict the performance of a scoring function on specific targets. This study seeks to address two questions. (1) Can employed rescoring strategies consistently improve discrimination binders from decoys? (2) Can the performance of docking and/or scoring be predicted by specification of the receptors binding sites?

## 2. Methods

### 2.1. Receptors and Ligand Preparation

32 diverse targets were selected from the DUD-E database [12] (Table 1). The selection was based on the diversity and size of the set to keep the computational cost as low as possible. The same 3D structures that had been used in DUD-E for each of the 32 selected targets were retrieved from protein data bank (PDB) (Table 1). Then, the PDB files were prepared for AutoDock Vina docking. Cocrystal ligands and water molecules were removed, hydrogen and partial charges (Gasteiger) were added, and the coordinates of the 3D structures were saved in pdbqt format. The ligands from the DUD-E data set were used following modifications. The ligands in the DUD-E set have been divided into active compounds and decoy compounds for each target. There are approximately 50 decoys for each active compound in the whole DUD-E set. The active group contained some duplicate structures that differ in their protonation states. As this would generate an analog bias, the duplicate forms were omitted, and only a single structure, which was in its physiological protonation state, was kept. The corresponding decoy structures were also omitted from the study. All the ligands were converted to pdbqt files. The number of active groups and decoys for each target were reported in Table 1.

### 2.2. Virtual Screening

The AutoDock Vina was employed for the molecular docking [5]. For each of the targets, a box was defined to dock the ligands properly in each active site. In all the docking runs, the exhaustiveness was set to 8. The cocrystal ligand for each target was redocked in the binding site of the target and the results are available as in Supplementary Materials (available here).

### 2.3. Rescoring

Four scoring functions and combinations of them have been evaluated in this study. These four scoring methods were from three different categories. Vina scoring (built-in scoring function of AutoDock Vina) and AutoDock4.2 scoring functions are force-field based. ChemScore is a SYBYL built-in scoring function that is an empirical scoring function. DrugScore is a knowledge base scoring function and is available as a standalone scoring function. All of the best docked poses of the ligands based on the Vina scoring function were rescored by other three scoring functions and also by all possible combinations. Thus, 11 consensus scorings were also applied (Tables 2 and 3).

A previously defined consensus scoring (rank-by-number method [9]) was employed to summarize the results of multiple scoring functions. Rank-by-number consensus score is an average of the *Z*-scaled scores calculated by each of the individual scoring functions. Individual *Z*-scaled scoring function values (*Z*Score) are computed by

(1) $$\begin{array}{c} Zscore=\frac{\left(fi-\mu \right)}{S}, \end{array}$$

where *fi* is the scoring value of an individual scoring function, *μ* is the mean value, and *S* is the standard deviation of this scoring function for entire set.

### 2.4. Calculation of Binding Site Descriptors

Binding site environment properties were retrieved form PLIC [18] database. This is a database that provides cluster of binding sites. It uses Fpocket [19] and LPC [20] to generate the following binding site descriptors: pocket volume, number of alpha spheres, mean alpha sphere radius, proportion of apolar alpha spheres, mean local hydrophobic density, hydrophobicity scores, volume score, charge score, proportion of polar atoms, alpha sphere density, maximum distance between COM and alpha sphere, Maximum Theoretical Shape Complementarity, observed shape complementarity, and normalized shape complementarity.

### 2.5. Statistical Analysis

To assess the performance of each scoring function and the consensus scoring two parameters were used: area under the curve (AUC) of the ROC (receiver operating characteristic) curve and enrichment factor (EF) at different levels. To evaluate the performance of the scoring functions in discriminating active groups among decoys the scoring functions performance was tested on docked active and decoy compounds. The ROC curve and EF were applied to determine the performance of each scoring function. The increase in AUC of the ROC curve can be used as an indicator of improvement in discrimination between true ligands from decoys. AUC can have a value between 0 and 1, in which AUC = 0.5 means that the method of interest performed like a random selection in average, while AUC = 1 means the complete discrimination between true and false cases (active and decoys). EF is defined as the fraction of active compounds found divided by the fraction of the screened library:

(2) $$\begin{array}{c} EF=\frac{actives_{sampled}}{actives_{total}}\times \frac{N_{total}}{N_{sampled}}. \end{array}$$

EF1% and EF2% showed the ability of a particular scoring method to retrieve true ligands with a high rank among virtual screening results.

Significance of the difference between the AUC of the two ROC curves was assessed using online tool at http://vassarstats.net/roc_comp.html. Other statistical tests and plotting were done using R (R: a language and environment for statistical computing; R Foundation for Statistical Computing, Vienna, Austria; URL http://www.R-project.org/) including the following packages: enrichvs and ROCR.

## 3. Results

**Figure 1:**
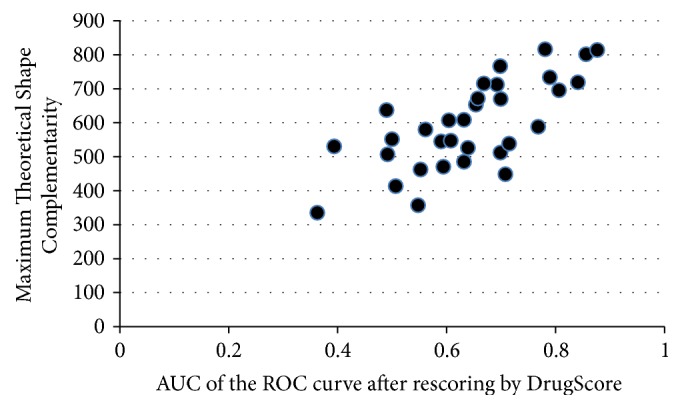
Significant correlation between performance of DrugScore and MTSC descriptor (correlation coefficient = 0.719, *p* value < 0.001).

**Figure 2:**
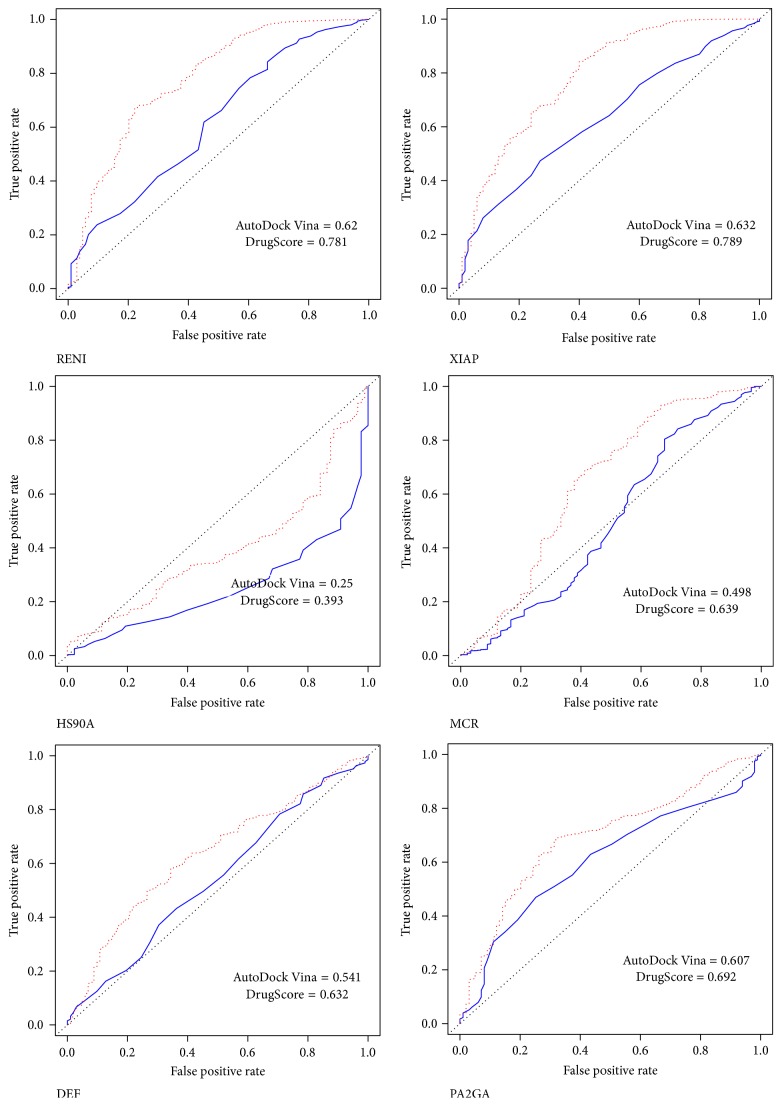
The cases with significant improvement in AUC of the ROC curves after rescoring with DrugScore (before: blue line; after: red dots).

**Figure 3:**
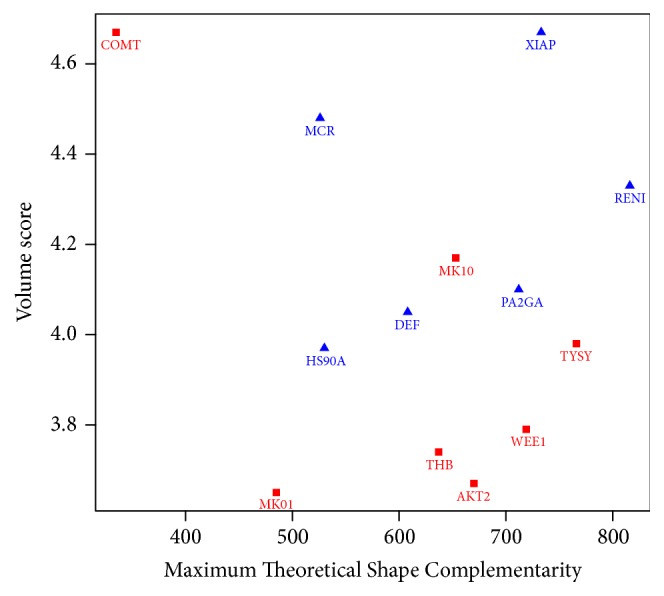
Separation of good and bad responders to DrugScore rescoring based on volume score and MTSC descriptors.

The average and difference in AUC of the ROC curve for each scoring method after rescoring are presented in Tables 2 and 3, respectively. They show the overall performance for each scoring method. The individual AUC of the ROC curve were shown in Table 4 and the details for each receptor and AutoDock Vina configuration files were presented in Supplementary Materials. The correlation between different scoring strategies and binding site descriptors was shown in Table 5. Screening power of AutoDock Vina original scoring and DrugScore demonstrated a good correlation with values of both Maximum Theoretical Shape Complementarity (MTSC) and Maximum Distance from Center of Mass and all Alpha spheres (MDCMA).  Figure 1 demonstrated this fair correlation between DrugScore performance and the binding site descriptor, MTSC. In Table 6 the protein targets whose AUC of the ROC curve were significantly increased or decreased after rescoring by DrugScore were emphasized (Figure 2). According to the various classifications plot (data not shown) it was found out that these two groups can be separated based on two descriptors, volume score and MTSC (Figure 3).

## 4. Discussion

The calculated performance of AutoDock Vina on individual target can be used for selection of this docking engine for virtual screenings on specific targets. Furthermore, the results showed slight general improvement in discrimination between decoys and ligands by using consensus rescoring method which consisted of Vina and DrugScore scoring functions. By active site analysis it was shown that DrugScore improved the discrimination power of AutoDock Vina significantly in case of receptors that had both high volume score and MTSC. In addition, it was shown that AutoDock and DrugScore Screening powers had significant correlation with MTSC and MDCMA.

AutoDock Vina is free for academics and has showed a good scoring power in a recent study on large and diverse data set [4]. Thus, it was selected as a docking engine for pose prediction in the present study. The screening power of AutoDock Vina was correlated with MTSC and MDCMA. The reported AUC of the ROC curve and enrichment factor could be used for prediction of AutoDock Vina performance on each target. Furthermore, MTSC and MDCMA values could be used as a possible indicator of successfulness of AutoDock Vina in a virtual screening on a specific target protein. It was suggested [21] that AutoDock Vina had a better average performance for 31 protein targets' virtual screening than DOCK [22]. As AutoDock Vina is an open source and shows good performance compared with other docking engines, improvements of AutoDock Vina code in different aspects such as parallel run [23] have been conducted during recent years.

It was suggested that the performances of docking program and scoring functions were target dependent [1, 4]. The nature of the active site of the proteins, the choice of scoring functions, and the set of ligands used for comparisons all affected the performance in scoring and ranking compounds [11]. Some studies concluded that consensus scoring (rank-by-number, consisting of three or four scoring functions) outperformed individual scoring performance [9]. In most of the studies that were conducted on more diverse and larger data sets, there is no strong correlation between affinity and scoring function predictions [4, 10]. In this study, only the ranking power of the scoring function was estimated. In overall consensus scoring with both DrugScore and Vina scoring functions, rescoring with DrugScore slightly improved the ranking metrics (AUC of the ROC curve and EF), but it was not statistically significant.

Rescoring by DrugScore produces most cases with significant increased or decreased screening power (assessed by changes in the AUC of the ROC curve) with respect to the original Vina scoring. Therefore, these data were used to find possible binding site descriptors that could predict the performance of DrugScore rescoring in improvement of original virtual screening results. Finally, after exploring different descriptors it was found that a simple model based on two descriptors (volume score and MTSC) could fairly predict the improvement of virtual screening results after rescoring by DrugScore for a target protein. DrugScore has been also successful in some other rescoring campaigns [8, 24] and was one of the best performers in a ranking power assessment among 16 scoring functions [7].

MTSC indicates the shape complementarity of a binding site with the specific cocrystalized ligand. Here, it was shown that the performance of DrugScore as well as AutoDock Vina docking and subsequent scoring are correlated with the value of MTSC. It could be due to the better performance of AutoDock Vina docking algorithm in finding near native pose of active groups in the case of a binding site with high MTSC. The values of the volume score descriptor were correlated with the improvement of virtual screening results by DrugScore rescoring. This could be explained as better performance of DrugScore in the case of the higher number of ligand-protein interactions in the bigger binding sites.

## 5. Conclusion

The results consistent with those previous studies suggested that performance of docking and scoring functions was target specific. Working on new scoring functions that include terms for aromatic-aromatic or *π*-cation or halogen protein interactions has been suggested. A correlation between screening power of AutoDock Vina and DrugScore and two binding site descriptors, MTSC and MDCMA, was found. The improvement after rescoring with DrugScore was predicted by two descriptors: volume score and MTSC. The ultimate goal of this study was to determine which of the scoring functions or combinations of them would yield the best results in terms of enrichment when used in a virtual screening study. The results could provide useful information for people to select the most appropriate target for using AutoDock Vina and/or DrugScore in their studies.

## Figures and Tables

**Table 1:** Data set characteristics.

Abbreviation used in DUD-E	Target name	PDB code	Number of ligands	Number of decoys
ADA	Adenosine deaminase	2E1W	93	5444
AKT2	Serine/threonine-protein kinase AKT2	3D0E	116	6891
COMT	Catechol O-methyltransferase	3BWM	41	3846
CP2C9	Cytochrome P450 2C9	1R9O	120	7435
CXCR4	C-X-C chemokine receptor type 4	3ODU	40	3406
DEF	*E. coli* peptide deformylase complexed with antibiotic actinonin	1LRU	102	5686
FA7	Coagulation factor VII	1W7X	114	6239
FKB1A	FK506-binding protein 1A	1J4H	111	5797
GLCM	Beta-glucocerebrosidase	2V3F	54	3799
GRIK1	Glutamate receptor ionotropic kainate 1	1VSO	101	6540
HS90A	Heat shock protein HSP 90-alpha	1UYG	88	4848
HXK4	Hexokinase type IV (human pancreatic glucokinase in complex with glucose and activator)	3F9M	91	4692
INHA	Enoyl-[acyl-carrier-protein] reductase (*Mycobacterium tuberculosis* enoyl reductase)	2H7L	43	2297
KIF11	Kinesin-like protein 1	3CJO	116	6844
KITH	Stem cell growth factor receptor (KIT kinase domain in complex with sunitinib)	2B8T	57	2850
MAPK2	MAP kinase-activated protein kinase 2	3M2W	101	6144
MCR	Mineralocorticoid receptor	2AA2	90	4835
MK01	MAP kinase ERK2	2OJG	79	4548
MK10	c-Jun N-terminal kinase 3 (mitogen-activated protein kinase 10)	2ZDT	104	6593
NOS1	Nitric-oxide synthase, brain	1QW6	100	8037
NRAM	Neuraminidase (influenza virus neuraminidase)	1B9V	98	6196
PA2GA	Phospholipase A2 group IIA	1KVO	99	5143
PLK1	Serine/threonine-protein kinase PLK1	2OWB	107	6794
PUR2	GAR transformylase	1NJS	50	2694
PYGM	Muscle glycogen phosphorylase	1C8K	77	3940
PYRD	Dihydroorotate dehydrogenase	1D3G	111	6443
RENI	Renin	3G6Z	104	6954
ROCK1	Rho-associated protein kinase 1	2ETR	100	6293
SAHH	Adenosylhomocysteinase	1LI4	62	3438
THB	Thyroid hormone receptor beta-1	1Q4X	103	7349
TYSY	Thymidylate synthase	1SYN	109	6732
WEE1	Serine/threonine-protein kinase WEE1	3BIZ	102	6135
XIAP	Inhibitor of apoptosis protein 3	3HL5	100	5143

**Table 2:** Average of AUC of the ROC curve and EF at different level obtained with each scoring approach (*v*: AutoDock Vina, *c*: ChemScore, *d*: DrugScore, and *a*: AutoDock 4.2).

Scoring	AUC	EF20%	EF10%	EF2%	EF1%	EF0.2%	EF0.1%
*v*	0.671	2.137	2.93	6.394	8.576	11.17	12.74
*c*	0.61	1.855	2.33	3.567	4.007	4.513	3.54
*d*	0.65	1.942	2.72	5.253	6.275	8.766	9.242
*a*	0.623	1.831	2.5	4.441	4.949	7.301	8.866
*vcda*	0.668	2.162	3.08	6.714	8.173	8.753	10.01
*vcd*	0.667	2.174	3.01	6.537	8.768	9.746	9.515
*vda*	0.677	2.2	3.21	7.096	9.169	11.72	14
*vca*	0.661	2.088	3.01	5.989	7.25	8.371	7.76
*cda*	0.652	2.068	2.84	5.158	6.086	7.41	7.47
*vc*	0.656	2.087	2.93	6.233	7.28	7.564	7.415
*vd*	0.679	2.14	3.08	7.026	9.292	13.21	14.76
*va*	0.671	2.192	3.05	6.074	8.562	11.72	15.66
*cd*	0.646	2.012	2.77	4.916	5.986	6.896	6.06
*ca*	0.631	1.895	2.53	4.322	5.057	7.324	6.865
*da*	0.658	2.026	2.85	5.579	7.182	8.688	8.51

**Table 3:** Average of difference between each rescoring approach in terms of AUC of the ROC curve and EF and original AutoDock Vina scoring (*v*: AutoDock Vina, *c*: ChemScore, *d*: DrugScore, and *a*: AutoDock 4.2).

Scoring	AUC	EF20%	EF10%	EF2%	EF1%	EF0.2%	EF0.1%
*v* − *v*	0.000	0.000	0.000	0.000	0.000	0.000	0.000
*c* − *v*	−0.061	−0.282	−0.600	−2.827	−4.569	−6.662	−9.201
*d* − *v*	−0.021	−0.195	−0.211	−1.140	−2.301	−2.409	−3.499
*a* − *v*	−0.048	−0.306	−0.432	−1.953	−3.627	−3.874	−3.875
*vcda* − *v*	−0.003	0.026	0.144	0.321	−0.403	−2.421	−2.736
*vcd* − *v*	−0.004	0.037	0.074	0.143	0.192	−1.429	−3.226
*vda* − *v*	0.006	0.063	0.281	0.702	0.593	0.541	1.263
*vca* − *v*	−0.010	−0.049	0.072	−0.405	−1.325	−2.804	−4.982
*cda* − *v*	−0.019	−0.068	−0.091	−1.236	−2.490	−3.765	−5.271
*vc* − *v*	−0.015	−0.050	−0.003	−0.161	−1.296	−3.611	−5.326
*vd* − *v*	0.008	0.004	0.147	0.632	0.716	2.033	2.014
*va* − *v*	0.000	0.055	0.119	−0.320	−0.014	0.543	2.920
*cd* − *v*	−0.025	−0.124	−0.167	−1.478	−2.590	−4.279	−6.681
*ca* − *v*	−0.040	−0.242	−0.399	−2.072	−3.519	−3.850	−5.877
*da* − *v*	−0.013	−0.111	−0.084	−0.814	−1.394	−2.487	−4.231

**Table 4:** AUC of the ROC curve obtained with each scoring method for individual targets (*v*: AutoDock Vina, *c*: ChemScore, *d*: DrugScore, and *a*: AutoDock 4.2; sorted based on AutoDock Vina performance).

	*v*	*c*	*d*	*a*	*vcda*	*vcd*	*vda*	*vca*	*cda*	*vc*	*vd*	*va*	*cd*	*ca*	*da*
WEE1	0.949	0.828	0.841	0.555	0.917	0.909	0.927	0.915	0.853	0.916	0.930	0.910	0.852	0.776	0.800
FA7	0.917	0.890	0.876	0.878	0.929	0.936	0.926	0.927	0.909	0.935	0.929	0.920	0.908	0.897	0.898
MAPK2	0.886	0.775	0.776	0.717	0.877	0.850	0.877	0.888	0.848	0.861	0.849	0.891	0.809	0.826	0.823
KIF11	0.858	0.845	0.806	0.840	0.860	0.860	0.856	0.865	0.846	0.867	0.852	0.864	0.842	0.849	0.835
TYSY	0.847	0.607	0.698	0.770	0.781	0.768	0.820	0.778	0.726	0.760	0.822	0.829	0.675	0.710	0.762
PYRD	0.826	0.749	0.768	0.730	0.791	0.807	0.795	0.784	0.767	0.803	0.817	0.789	0.778	0.747	0.763
PUR2	0.819	0.393	0.856	0.691	0.749	0.762	0.827	0.667	0.702	0.641	0.869	0.777	0.696	0.557	0.801
MK01	0.806	0.767	0.632	0.629	0.748	0.777	0.719	0.774	0.702	0.816	0.747	0.748	0.726	0.721	0.639
AKT2	0.778	0.744	0.699	0.803	0.801	0.788	0.810	0.794	0.795	0.776	0.786	0.806	0.765	0.785	0.799
THB	0.777	0.484	0.490	0.578	0.632	0.630	0.665	0.700	0.504	0.693	0.670	0.777	0.480	0.523	0.510
MK10	0.746	0.701	0.653	0.598	0.694	0.721	0.682	0.697	0.666	0.737	0.716	0.684	0.692	0.659	0.633
FKB1A	0.693	0.755	0.657	0.668	0.730	0.745	0.697	0.734	0.724	0.755	0.702	0.690	0.738	0.736	0.676
INHA	0.688	0.680	0.715	0.693	0.719	0.722	0.719	0.708	0.712	0.705	0.723	0.702	0.712	0.696	0.714
KITH	0.688	0.532	0.699	0.621	0.646	0.655	0.667	0.628	0.632	0.631	0.692	0.654	0.636	0.592	0.658
SAHH	0.677	0.290	0.708	0.615	0.590	0.575	0.719	0.516	0.539	0.478	0.726	0.685	0.512	0.391	0.694
ROCK1	0.666	0.660	0.594	0.654	0.668	0.662	0.659	0.678	0.657	0.680	0.642	0.674	0.645	0.666	0.641
CXCR4	0.661	0.726	0.604	0.723	0.706	0.687	0.685	0.729	0.706	0.718	0.640	0.712	0.682	0.735	0.681
XIAP	0.632	0.676	0.789	0.678	0.724	0.739	0.722	0.681	0.741	0.669	0.741	0.668	0.772	0.694	0.742
RENI	0.620	0.686	0.781	0.588	0.694	0.733	0.688	0.638	0.707	0.664	0.742	0.605	0.759	0.642	0.703
PLK1	0.619	0.628	0.668	0.548	0.628	0.653	0.625	0.605	0.625	0.629	0.659	0.588	0.659	0.592	0.620
CP2C9	0.613	0.604	0.552	0.563	0.597	0.607	0.588	0.605	0.582	0.622	0.593	0.597	0.588	0.587	0.564
PA2GA	0.607	0.795	0.692	0.814	0.791	0.760	0.768	0.783	0.812	0.746	0.696	0.744	0.771	0.821	0.801
PYGM	0.594	0.597	0.561	0.446	0.561	0.597	0.543	0.555	0.540	0.608	0.583	0.530	0.588	0.522	0.502
NOS1	0.570	0.551	0.506	0.492	0.545	0.545	0.533	0.570	0.533	0.570	0.533	0.569	0.533	0.551	0.506
DEF	0.541	0.262	0.632	0.578	0.502	0.465	0.602	0.456	0.485	0.384	0.602	0.569	0.427	0.415	0.621
GRIK1	0.538	0.464	0.492	0.442	0.483	0.500	0.493	0.480	0.460	0.503	0.518	0.495	0.480	0.439	0.467
NRAM	0.526	0.522	0.608	0.443	0.537	0.574	0.537	0.496	0.537	0.530	0.581	0.478	0.589	0.481	0.536
COMT	0.525	0.371	0.363	0.736	0.575	0.398	0.645	0.646	0.593	0.431	0.439	0.750	0.340	0.688	0.690
ADA	0.520	0.377	0.500	0.435	0.438	0.444	0.479	0.428	0.417	0.430	0.509	0.474	0.416	0.395	0.459
HX4	0.515	0.552	0.590	0.534	0.550	0.554	0.549	0.533	0.563	0.532	0.555	0.524	0.573	0.545	0.566
MCR	0.498	0.656	0.639	0.563	0.628	0.634	0.571	0.589	0.691	0.584	0.571	0.495	0.699	0.665	0.634
GLCM	0.486	0.471	0.548	0.541	0.520	0.506	0.536	0.506	0.528	0.484	0.517	0.520	0.515	0.507	0.559
HS90A	0.250	0.321	0.393	0.369	0.308	0.290	0.295	0.316	0.346	0.270	0.296	0.294	0.338	0.310	0.378

**Table 5:** Pearson correlation coefficients between each rescoring approach and binding site descriptors (*v*: AutoDock Vina, *c*: ChemScore, *d*: DrugScore, and *a*: AutoDock 4.2).

	Pocket volume	Number of alpha spheres	Mean alpha sphere radius	Proportion of apolar alpha spheres	Mean local hydrophobic density	Hydrophobicity score	Volume score	Charge score	Proportion of polar atoms	Alpha sphere density	Max Dist. from Center of Mass and all Alpha spheres	Maximum Theoretical Shape Complementarity	Observed shape complementarity	Normalized shape complementarity
*v*	0.415	0.418	−0.055	0.001	0.249	−0.108	−0.190	0.170	0.098	0.458	0.594	0.532	0.439	0.085
*c*	0.509	0.400	0.068	0.126	0.266	0.131	−0.153	0.320	−0.204	0.445	0.459	0.462	0.304	−0.042
*d*	0.510	0.387	0.111	−0.066	0.161	−0.011	0.014	0.171	0.141	0.366	0.555	0.719	0.474	−0.022
*a*	0.234	0.355	0.034	0.095	0.217	0.103	0.135	0.268	−0.118	0.207	0.378	0.413	0.303	0.030
*vcda*	0.483	0.424	0.061	0.058	0.274	0.001	−0.057	0.307	−0.041	0.419	0.586	0.607	0.415	−0.005
*vcd*	0.541	0.471	0.064	0.031	0.276	0.017	−0.142	0.274	−0.011	0.483	0.620	0.665	0.464	0.000
*vda*	0.417	0.387	0.043	0.010	0.234	−0.081	−0.011	0.243	0.051	0.363	0.573	0.588	0.425	0.035
*vca*	0.443	0.410	0.028	0.104	0.295	0.011	−0.083	0.328	−0.105	0.425	0.557	0.515	0.365	0.005
*cda*	0.476	0.378	0.085	0.080	0.244	0.068	0.028	0.334	−0.095	0.361	0.519	0.582	0.356	−0.056
*vc*	0.515	0.468	0.031	0.073	0.302	0.022	−0.202	0.292	−0.073	0.507	0.596	0.562	0.413	0.015
*vd*	0.495	0.454	0.031	−0.025	0.241	−0.074	−0.112	0.187	0.112	0.453	0.631	0.684	0.506	0.045
*va*	0.331	0.354	−0.012	0.043	0.241	−0.089	−0.036	0.247	0.011	0.349	0.532	0.459	0.365	0.064
*cd*	0.554	0.426	0.100	0.033	0.234	0.087	−0.086	0.292	−0.048	0.439	0.553	0.651	0.409	−0.058
*ca*	0.412	0.339	0.061	0.148	0.250	0.116	0.022	0.346	−0.212	0.337	0.436	0.439	0.272	−0.047
*da*	0.379	0.336	0.075	0.023	0.184	−0.002	0.143	0.269	0.009	0.260	0.479	0.567	0.366	−0.017

**Table 6:** Difference between calculated AUC of the ROC curve after rescoring with DrugScore and original AUC of the ROC curve for each target (^*∗*^statistically significant changes).

Receptor	*d* − *v*
THB^*∗*^	−0.2876
MK01^*∗*^	−0.1738
COMT^*∗*^	−0.1625
TYSY^*∗*^	−0.1491
WEE1^*∗*^	−0.1083
MK10^*∗*^	−0.0929
AKT2^*∗*^	−0.0787
ROCK1	−0.0715
NOS1	−0.0641
CP2C9	−0.0605
PYRD	−0.0582
CXCR4	−0.0569
KIF11	−0.0518
GRIK1	−0.0467
FA7	−0.0403
FKB1A	−0.036
PYGM	−0.0327
ADA	−0.02
KITH	0.0107
INHA	0.0262
SAHH	0.0303
PUR2	0.0364
PLK1	0.0492
GLCM	0.062
HX4	0.0753
NRAM	0.0812
PA2GA^*∗*^	0.0854
DEF^*∗*^	0.0906
MCR^*∗*^	0.1411
HS90A^*∗*^	0.1439
XIAP^*∗*^	0.1577
RENI^*∗*^	0.1605

## Supplementary Materials

Supplementary Materials Supplementary Materials contain a folder (configuration files) which includes the configuration files of the AutoDock Vina, a Microsoft Excel file (RMSD-redocking) which includes all of the RMSD obtained after redocking the cocrystal ligands in each corresponding target active site, and another Microsoft Excel file (rescoring-details) which includes detailed results of the rescoring study on each protein target.
